# Suppurative Pericarditis

**Published:** 1901-09-14

**Authors:** 


### SUPPURATIVE PERICARDITIS.

Although pericarditis in children is most often a manifestation of rheumatism there are other forms which, although rare in comparison with the rheumatic affection, are probably less rare in childhood than they are at other times of life. Among them may be mentioned that form of pericarditis which is found in association with suppurative conditions elsewhere, and is due to infection of the pericardium with pyogenic micro-organisms. Dr. Still, speaking on this subject at the annual meeting of the British Medical Association, said that the term suppurative pericarditis was not altogether satisfactory, for the pericardial fluid was hardly sufficiently turbid in some cases to be called purulent. Still these cases were the result of pyogenic infection, and for an accurate conception of suppurative pericarditis it was necessary to take into consideration not only the cases of fully-developed suppuration, with thick opaque pus, but also those cases in which the disease was in an early stage and the pericardial fluid was only slightly clouded. Suppurative pericarditis is much more likely to occur in infants than in the later half of childhood. It is almost always part of a pneumococcal infection which has in most cases begun with pneumonia, and has affected the pleura, and it is to be noted that the presence of lymph or of turbid serum in the pleura involves a risk of suppurative pericarditis no less than does an actual empyema. Such an infection of the pericardium may occur within a few days after the onset of the pneumonia or not until several weeks have elapsed, and only the empyema or the pleurisy remains. In any case, therefore, where exploration of the chest shows turbid serum or pus, especially if on bacteriological examination the fluid is found to contain pneumococci, free drainage, by resection of a rib in most cases, should be provided with as little delay as possible; and this should be done, however small the accumulation may seem to be, for so long as such infective material remains shut up in the chest the child is in hourly risk of further infection, which, should it involve the pericardium or the meninges, is likely to prove fatal. In the diagnosis of this condition it is worth remembering that there is usually no great quantity of fluid in the pericardium. This fact, together with the absence or slightness of cardiac dilatation —so different from what occurs in rheumatism—accounts for the very slight increase of cardiac dulness which is observed in some of these cases. Again, it is to be observed that occasionally very extensive adhesions of the pericardium may form, so that an exploratory puncture in the usual position may give a negative result. For this reason exploratory incision after removal of a portion of one of the costal cartilages just to the left of the sternum seems to be a much more satisfactory method of exploration if circumstances make it possible. Lastly, as to the possibility of recovery in a child with this form of pericarditis, it is clear that with timely drainage life may be saved even though the pericardial fluid has become actually purulent. But in many cases the presence of other lesions arising from a general infection makes the prognosis very bad.

